# Mitigation of Ergot Vasoconstriction by Clover Isoflavones in Goats (*Capra hircus*)

**DOI:** 10.3389/fvets.2016.00017

**Published:** 2016-03-04

**Authors:** Glen E. Aiken, Michael D. Flythe, Isabelle A. Kagan, Huihua Ji, Lowell P. Bush

**Affiliations:** ^1^USDA Agricultural Research Service Forage-Animal Production Research Unit, Lexington, KY, USA; ^2^Kentucky Tobacco Research and Development Center, University of Kentucky, Lexington, KY, USA; ^3^Plant and Soil Sciences Department, University of Kentucky, Lexington, KY, USA

**Keywords:** ergot alkaloids, goats, isoflavones, tall fescue, vasoconstriction

## Abstract

Ergot alkaloids produced by a fungal endophyte (*Epichloë coenophiala*; formerly *Neotyphodium coenophialum*) that infects tall fescue (*Lolium arundinaceum*) can induce persistent constriction of the vasculature in ruminants, hindering their capability to thermo-regulate core body temperature. There is evidence that isoflavones produced by legumes can relax the vasculature, which suggests that they could relieve ergot alkaloid-induced vasoconstriction and mitigate the vulnerability to severe heat stress in ruminants that graze tall fescue. To test if isoflavones can relieve alkaloid-induced vasoconstriction, two pen experiments were conducted with rumen-fistulated goats (*Capra hircus*) to determine with ultrasonograpy if isoflavones can (1) promote vascular compliance by countering alkaloid-induced vasoconstriction and (2) relieve already imposed alkaloid-induced vasoconstriction. Goats were fed *ad libitum* chopped orchardgrass (*Dactylis glomerata*)–timothy (*Phleum pratense*) hay prior to conducting the experiments. Measures of carotid and interosseous luminal areas were obtained pre- (baseline) and post-ruminal infusions in both experiments with goats being fed the hay, and for blood flow rate in the carotid artery in Experiment 2. Responses to infusion treatments were evaluated as proportionate differences from baseline measures. Peak systolic velocity, pulsatility index, and heart rate were measured on the last day on treatment (DOT) in Experiment 1, and on all imaging sessions during Experiment 2. For Experiment 1, rumens were infused with ground toxic fescue seed and isoflavones in Phase A and with only the toxic seed in Phase B. The infusion treatments were switched between phases in Experiment 2, which employed a fescue seed extract having an ergot alkaloid composition equivalent to that of the ground seed used in Experiment 1. During Experiment 1, luminal areas of carotid and interosseous arteries in Phase A did not deviate (*P* > 0.1) from baselines over 1, 2, 3, and 4 DOT, but the areas of both declined linearly from baselines over 1, 2, 3, and 4 DOT in Phase B. By 6, 7, and 8 DOT in Experiment 2, luminal areas of the arteries and flow rate declined from baselines with infusions with the only seed extract in Phase A, but luminal areas and flow rate increased over 4, 5, and 6 DOT with the additional infusion of isoflavones. Peak systolic velocity and heart rate were not affected by treatment in either experiment, but were highest when infused with only ergot alkaloids in both experiments. Treatment with isoflavones was demonstrated to relax the carotid and interosseous arteries and reduce resistance to blood flow. Results indicate that isoflavones can relax persistent vasoconstriction in goats caused by consumption of ergot alkaloids, and mitigate the adverse effect that ergot alkaloids have on dry matter intake.

## Introduction

Tall fescue (*Lolium arundinaceum*) is a cool-season perennial grass that covers ~15 million hectares in the eastern half of the USA and is extensively utilized for livestock grazing. The grass is productive and tolerant of environmental stresses, which has been attributed to the presence of a fungal endophyte (*Epichloë coenophiala*) that infects most plants of tall fescue (1). However, ergot alkaloid toxins are also produced by the endophyte, and they cause a toxicosis in grazing livestock. Signs of “fescue toxicosis” include poor weight gain and reproductive performances, elevated body temperature, labored respiration, and decreased serum prolactin (2, 3).

Ergot alkaloids bind biogenic amine receptors in the vasculature (4) to induce persistent vasoconstriction (5) that incapacitates the animal’s ability to regulate core body temperature (6, 7). Cattle (5, 8), sheep (9), horses (10), and goats (11) have been reported to vasoconstrict when exposed to ergot alkaloids. Therefore, the vasculatures of these livestock species are vulnerable to ergot alkaloid-induced vasoconstriction. Ergot alkaloids also bind D2 receptors of the anterior pituitary gland to reduce secretion of prolactin (3, 4).

Isoflavones are phytoestrogens commonly found in legumes and can have estrogenic activity in mammals (12). Isoflavones have been used as a treatment for ameliorating hot flashes in postmenopausal women (13, 14). Cruz et al. (15) used *in vitro* techniques with small arteries collected from subcutaneous circulation of men with and without coronary heart disease to determine a vaso-relaxation response with exposure to phytoestrogens, resveratrol and genistein. Relaxation of the vasculature from exposure to isoflavones has been associated with agonist activity of isoflavones at β-adrenergic receptors in the vasculature endothelium to stimulate synthesis of nitric oxide that mediates vaso-relaxation (13–16). Recently, Shappell et al. (17) reported an additive effect of combining estradiol implants with feeding soybean hulls on the estrogenic activity of serum from steers grazing toxic tall fescue. Although it could not be concluded, the mitigation of fescue toxicosis observed with this treatment combination (18) could have been related to estrogenic activity with the treatment combinations being above a threshold for relief of vasoconstriction to occur. It could be possible that β-agonist activity of isoflavones could ameliorate the persistent vasoconstriction caused by ergot alkaloids. Therefore, a pen study was conducted using color Doppler and B-mode ultrasonography to determine if treatment of wether goats with isoflavones can mediate relaxation of their carotid and interosseous arteries during and after the occurrence of ergot alkaloid-induced vasoconstriction.

## Materials and Methods

Two independent experiments were conducted in the basement of the Garrigus Building on the University of Kentucky Campus using in-door pens maintained at ambient temperature of ~21°C. The same set of six Spanish, wether goats that were ~2-year old and with rumen fistulas were used in both experiments. Initial body weights for Experiments 1 and 2 averaged 36.3 ± 2.6 (SD) and 38.6 ± 5.0 kg, respectively. Goats were weighed initially and periodically to make sure that body weights of the mature goats were stable during the experiment. Handling of goats and data collection followed procedures approved by the University of Kentucky Institutional Animal Care and Use Committee (protocol number 2013-1152).

With the low number of fistulated goats available for the study and the possibility of amelioration of ergot alkaloid-induced vasoconstriction being subtle and over a long period of time, it was decided to keep all goats on the same infusion treatment within a phase for each experiment. Carry-over effects of ergot alkaloids are not understood, so the goats were kept on the non-toxic hay for 78 days between the two experiments. Furthermore, conduct of the experiments in a controlled environment (21 ± 1°C) minimized confounding effects of time and changes in environmental conditions.

### Isoflavone Analysis

Promensil^®^ (Natrol Inc., Chatsworth, CA, USA), an over-the-counter red clover dietary supplement that has been safely administered to humans in clinical studies (19) was infused trans-ruminally as a source of isoflavones.

For assaying isoflavones in the red clover tablets (~600 mg), tablets were incubated in 7.5 mL water in a 50-mL tube for about 30 min (including 10 min at 27–32°C) to dissolve other additives in the tablets. After a brief centrifugation (3 min, 25°C, 2200 × *g*), the supernatant was discarded. Loss of isoflavones in this first step was expected to be minimal because preliminary extractions in water had demonstrated that after 1–2 h of stirring or sonicating, ~2.3 mg of isoflavones (6% of the expected total) was recovered.

The pellet recovered from the above centrifugation was then extracted after the method of Flythe and Kagan (20), but with volumes modified for the larger samples, and with some heating to approximate changes that might occur in the rumen. The pellet remaining after the water incubation was sonicated for 30 min, 28–35°C, in 8.4 mL 85% MeOH in 0.5% aqueous acetic acid. This extraction solvent included an internal standard, consisting of 0.58 mM catechin (Sigma-Aldrich, St. Louis, MO, USA) prepared in MeOH. After addition of 3.6 mL water (final solvent composition of 60% MeOH in 0.35% aqueous acetic acid), samples were centrifuged (8 min, 25°C, 2200 × g,), and 5 mL supernatant was filtered through a 0.45-μm hydrophilic membrane (GH Polypropylene, Pall Life Sciences, Ann Arbor, MI, USA). A portion of filtrate was diluted threefold in the same methanol–acetic acid solvent (to be within the range of the standard curve) and used for high-performance liquid chromatography (HPLC) analysis. Diluted extracts were separated on an Agilent 1100 HPLC system equipped with a Merck LiChrospher RP-18 endcapped column (250 mm length × 4.6 mm i.d.), using separation conditions described previously (21). Isoflavones were monitored at 270 nm and quantified based on calibration curves of standards purchased from Sigma-Aldrich (biochanin A) or Indofine Chemical (genistein). Aglycones and their corresponding glycosides, if identifiable, were summed to give the total amounts of each isoflavone of interest. Formononetin was quantified based on a biochanin A calibration curve, which decreased the final formononetin concentrations by about 20% and, thus, gave a slightly more conservative number. Genistein was quantified with a genistein calibration curve. Genistin, the genistein glucoside, was not included in the genistein quantification because it was not detectable at the concentrations injected. Similarly, daidzein was not detected.

The amounts of biochanin A, formononetin, and genistein (representing the sum of aglycones and corresponding glycosides if present) were corrected for recovery, based on the amount of catechin recovered in extracts. Recoveries were about 80%. When biochanin A, formononetin, and genistein content were averaged among six replicates, the mean concentrations per tablet, with the extraction and analytical methods used, were 5.7 ± 0.7 mg biochanin A, 5.2 ± 1.0 mg formononetin, and 0.42 ± 0.05 mg genistein. These results did not include the contribution from the biochanin A malonylglucoside, and they underestimated the amount of formononetin by about 20% because formononetin was quantified with a biochanin A calibration curve, which has a higher response factor.

### Infusion Treatments

Rumen infusion treatments were evaluated in each experiment. Goats were penned as pairs and were adapted to *ad libitum* consumption of a chopped orchardgrass (*Dactylis glomerata*)–timothy (*Phleum pratense*) hay for a minimum of 35 days prior to initiation of the infusion treatments. Quantity of Promensil infused daily (~27 g) was set to provide 30 mg/L of biochanin A, the isoflavone of highest concentration in the red clover tablets. In Experiment 1, ground toxic endophyte-infected seed of “Kentucky 31” tall fescue and isoflavones were infused in combination into the rumen of each goat during the Phase A of the experiment. Seed was ground using a Wiley Mill with 5.0 mm screen. Quantities of ground seed infused daily were targeted to provide a diet concentration of ~0.8 μg/g of diet DM of ergovaline plus its epimer, ergovalinine. Amounts of seed were daily adjusted based on the previous day’s consumption for each goat pair. In Experiment 2, toxic seed was replaced with an extract of toxic endophyte-infected tall fescue that was generated following procedures described by Foote et al. (22). The extract was wrapped in a soft tortilla for infusion in a quantity that targeted a diet concentration of 1.1 μg ergovaline/ergovalinine per g DM of hay. Ground seed used in Experiment 1 and seed extract used in Experiment 2 were from composite of seed samples from bags of the endophyte-infected cultivar, “Defiance.” Seed and extract were analyzed for concentrations of the ergopeptine, ergovaline, and its epimer, ergovalinine, using procedures of Yates and Powell (23) and modified as described by Carter et al. (18). Concentrations of ergovaline/ergovalinine in the seed averaged 2.33 μg/g, and the amount in daily fed seed extract per goat was 722.0 μg.

Chopped orchardgrass–timothy hay was fed *ad libitum*. Seed that was previously coarse-ground using a Wiley Mill with a 5.0-mm screen was infused into each rumen using a funnel. Feeding of hay and rumen infusions were done at 1500 hours each day. During the experimental periods, the previous day’s intake for each pen was used to estimate intake per goat and the ratio of fed chopped hay to rumenally infused seed for providing a daily diet concentration of 0.8 μg of ergovaline and ergovalinine, per gram of dry matter. Because a 30 ppm dose of biochanin A was found to decrease ammonia production in mixed goat rumen bacteria *in vitro* (24), the amount of the Promensil extract needed for each goat was based on the amount needed to achieve a biochanin A concentration of 30 mg per liter of rumen. The volume of the goat rumen was estimated at 8.5 L.

### Experiment 1

The goats were fed chopped orchardgrass–timothy hay *ad libitum* for 35 days prior to start of infusion treatments. During Phase A of the experiment, goats were infused daily with endophyte-infected seed and isoflavones in amounts that were previously discussed. Phase B was conducted with infusion of only the toxic ground seed for a 5-day duration.

Color Doppler ultrasound images of the cross-sections of the left common carotid artery and left recurrent interosseous artery over the scapula-humerus joint were collected using a Classic Medical TeraVet 3000 Ultrasound Unit (Classic Universal Ultrasound, Tequesta, FL, USA) with a 12L5-VET (12 MHz) linear array transducer. Hair was kept clipped with surgical clippers at each site to maintain consistent transducer placement at each imaging session. Baseline measures for hay feeding with no rumen infusions were collected for each goat on the day before and the fourth and fifth days prior to start of infusions for the Phase A. Images were collected on days 1, 2, 3, and 4, and during Phase B on days 1, 2, 3, 4, and 5. Each imaging session was started at ~1100 hours and was done in 30–40 min. Five images were collected and stored for each artery using a pulse frequency of 5.0 MHz. Scan depth was set at 4 cm for the common carotid artery and 3 cm for the recurrent interosseous and caudal arteries. Following freezing of an individual scan, frames stored in the cine memory of the unit were searched to store the image exhibiting the maximum flow signal and assumed to be at peak systolic phase. The flow signal was traced to estimate lumen area (8).

On the final day on treatment (DOT) for each infusion treatment, five longitudinal images of the left common carotid arteries also were collected using Pulse Wave Doppler feature to measure peak systolic velocity, heart rate, and pulsatility index. Pulsatility index was used as an indicator of vascular resistance (25). Images were collected on the last DOT that cross-sectional images were collected for the two infusion treatments, but not for baseline measures. Doppler spectra were collected using a Doppler frequency of 5.0 MHz, a correction angle of 60°, and a sample volume size of 1.0 mm.

### Experiment 2

The goats were fed chopped orchardgrass–timothy hay *ad libitum* for 77 days prior to start of infusion treatments. The seed extract was rumenally infused daily into each goat for 8 days during Phase A, and both the seed extract and isoflavones were infused for 6 days during Phase B.

Ultrasound B-mode images of the cross-sections of the left common carotid and left recurrent interosseous arteries were collected with a Z. One Ultra SP System (Zonare Medical Systems, Inc., Mountain View, CA, USA) using the L14-5W linear array transducer. Baseline measures for hay feeding with no rumen infusions were collected for each goat on the same days prior to initiation of the infusion treatment as for Experiment 1. Another difference from Experiment 1 was that images were collected on latter days during each phase when, based on observations in Experiment 1, a response was assumed to be present. Images were collected during Phase A on days 6, 7, and 8, and during Phase B on days 4, 5, and 6. The B-mode images were collected using a frequency of 5.0 MHz. Five image clips were stored for each artery for freezing the still image exhibiting the maximum flow signal and assumed to be at peak systolic phase for measurement of lumen diameter and calculation of luminal area. The elastin tissue in the endothelial intima was used for tracing the lumen and estimating luminal area (8).

Similar to Experiment 1, five longitudinal images of the left common carotid arteries also were collected using the Pulse Wave Doppler feature to measure peak systolic velocity, mean velocity, heart rate, and pulsatility index; however, unlike Experiment 1, the images were collected when baseline cross-sectional images were collected, and after initiation of infusion treatments on DOT when cross-sectional images were collected. Instrument settings were the same between both experiments.

### Statistical Analyses

Proportionate differences of luminal areas of arteries during the phases from baseline measures were analyzed using mixed models of SAS as repeated measures with the autoregressive covariance (26). The analysis used individual animals as the experimental unit. Infusion treatment, DOT, and the interaction between infusion treatment and DOT were analyzed as fixed effects for both experiments, and goat and goat × fusion treatment were evaluated as random effects. Measures from images collected when hay was fed with no infusions were averaged for each goat and subtracted from measures made during the two phases of each experiment, and divided by the average baseline measure to estimate the proportionate difference. Infusion treatment was analyzed as a discrete variable and DOT was evaluated as a continuous variable. In the presence of interaction between infusion treatment and DOT, least square means for luminal areas in both experiments and blood flow rate in the carotid artery measured in Experiment 2 were evaluated as being above, below, or similar (*P* < 0.05) to baseline measures. Linear trends over DOT for each infusion treatment were determined as described by Littell et al. (26) using mixed models.

Blood flow characteristics for the carotid were analyzed using the same statistical model as the artery luminal areas, with the exception that actual measures and not proportionate differences were analyzed. The PDIFF option was used for comparisons of least square means between baselines and the two infusion treatments in Experiment 2.

## Results

### Experiment 1

**Figure 1:**
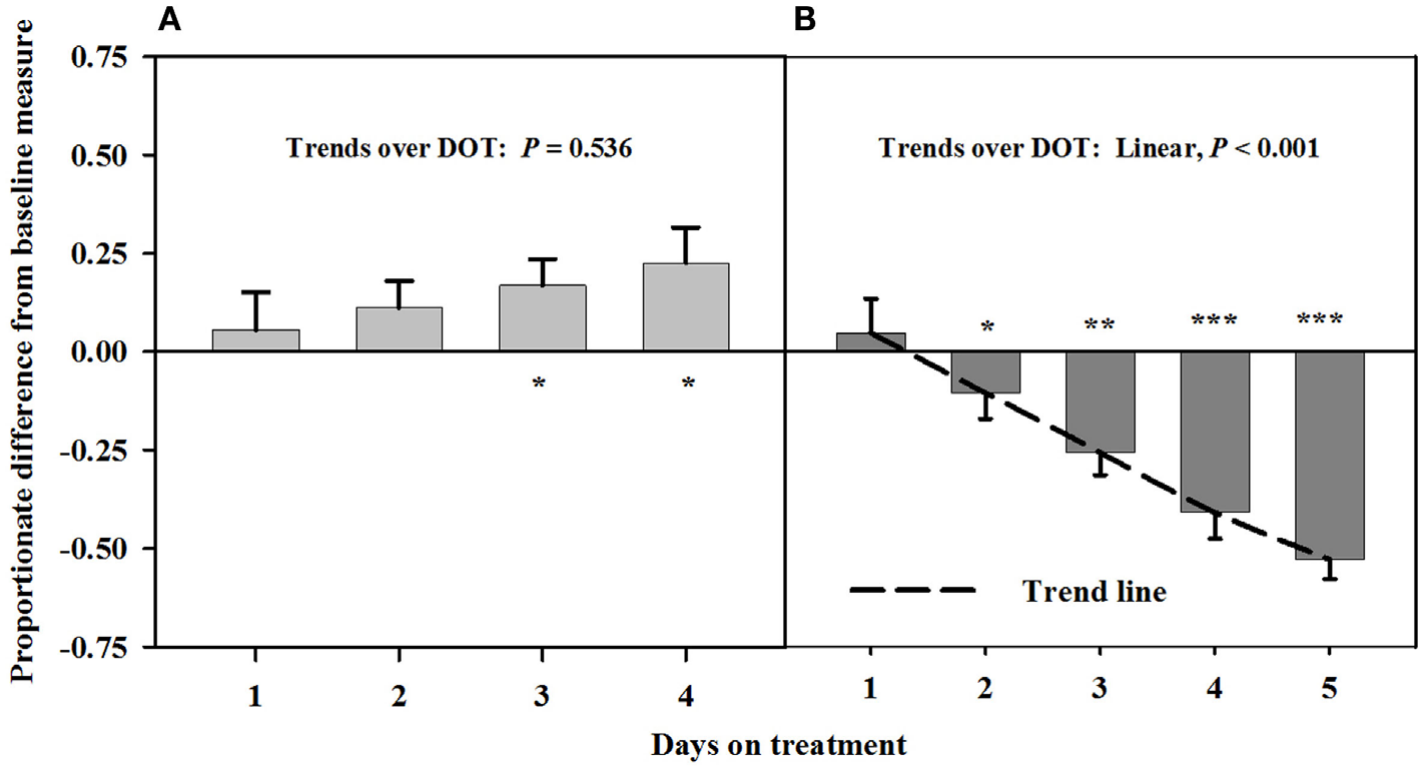
**Ultrasonic measures in Experiment 1 of proportionate differences from baseline measures for luminal area of the carotid artery in rumen-fistulated wether goats that were infused with (A) toxic endophyte-infected tall fescue seed and isoflavones in phase A, and (B) toxic seed only in phase B**. Baseline measures were taken with goats receiving *ad libitum* chopped orchardgrass (*Dactylis glomerata*)–timothy (*Phleum pratense*) hay with no rumen infusions. Images for determining baseline measures were collected when goats were being adapted to the hay diet on the last, fourth, and fifth days prior to initiating the infusion treatments. Asterisks above the bars denote significant differences between treatment and baseline measures at *P* < 0.05 (*), *P* < 0.01 (**), and *P* < 0.001 (***) levels of significance. A trend line is provided for Phase B that illustrates the linear relationship between proportionate differences from baseline and days on treatment.

Baseline measures of the carotid luminal areas averaged 12.1 ± 0.7 mm^2^. There were no trends over DOT during Phase A with infusions of toxic seed and isoflavones. However, carotid luminal areas were similar (*P* > 0.12) to baselines for the first 2 days, but were greater (*P* < 0.05) than baselines on days 3 and 4 (Figure 1). During infusion with only the toxic seed in Phase B, there was a linear decline in carotid luminal area over DOT. Mean luminal area fell below the baseline by day 3 (26% decrease from the baseline measure) and remained proportionately less on day 4 (45% decrease from the baseline measure).

**Figure 2:**
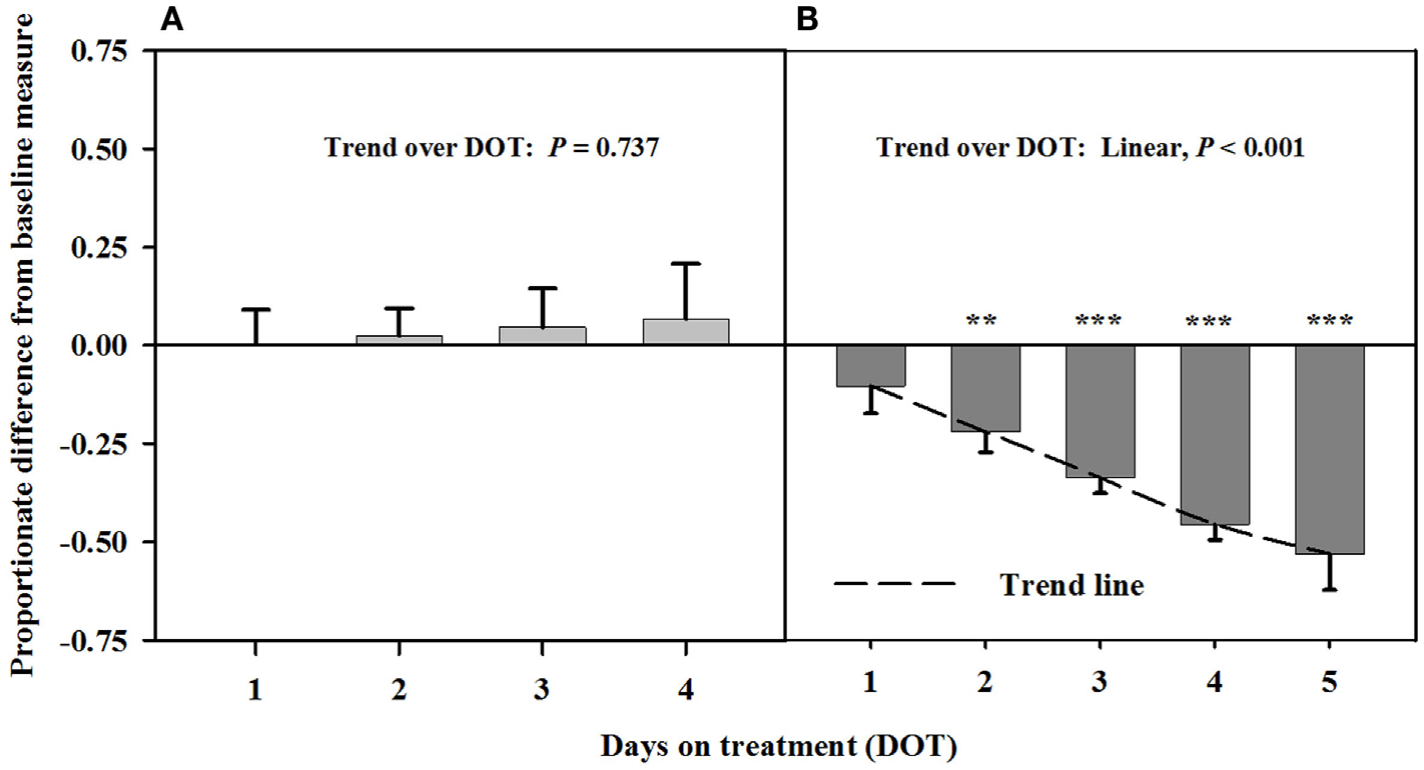
**Ultrasonic measures in Experiment 1 of proportionate differences from baseline measures for luminal area of the interosseous artery in rumen-fistulated wether goats that were infused with (A) toxic endophyte-infected tall fescue seed and isoflavones in phase A, and (B) toxic seed only in phase B**. Baseline measures were taken with goats receiving *ad libitum* chopped orchardgrass (*Dactylis glomerata*)–timothy (*Phleum pratense*) hay with no rumen infusions. Images for determining baseline measures were collected when goats were being adapted to the hay diet on the last, fourth, and fifth days prior to initiating the infusion treatments. Asterisks above the bars denote significant differences between treatment and baseline measures at *P* < 0.05 (*), *P* < 0.01 (**), and *P* < 0.001 (***) levels of significance. A trend line is provided for Phase B that illustrates the linear relationship between proportionate differences from baseline and days on treatment.

Interosseous luminal areas (mean baseline measure = 2.7 ± 0.1 mm^2^) with infusions of toxic seed and isoflavones during Phase A did not differ from baseline measures, but were increasingly less than the baseline over DOT during Phase B when only the toxic seed was infused (Figure 2). Mean luminal areas were proportionately less than baselines on 2, 3, and 4 DOT during Phase B, with ranges in areas relative to the baseline of 22% at 2 DOT to 45% at 4 DOT.

There were no differences between the infusion treatments (Table 1) on peak systolic velocity (*P* = 0.47) or heart rate (*P* = 0.712). A greater pulsatility index occurred at the conclusion of Phase B, during which only toxic seed was infused, which indicated greater resistance to blood flow with infusion of ergot alkaloids and less resistance to blood flow when isoflavones were combined with the toxic seed in the infusion.

**Table 1:** **Mean responses (±SEM) of blood flow characteristics in rumen-fistulated wether goats in Experiment 1 when toxic tall fescue seed and isoflavones were rumenally infused in Phase A and only toxic tall fescue seed was infused in Phase B, and in Experiment 2 when toxic seed extract only was infused in Phase A and toxic seed extract and isoflavones were infused in Phase B**.

Experiment	Measure	Baseline	Phase A	Phase B
1	Peak systolic velocity, cm/s		29.8 ± 3.6	33.2 ± 2.6
	Pulsatility index		1.41 ± 0.18^b^	3.39 ± 0.71^a^
	Heart rate, bpm		61 ± 4	63 ± 3
2	Peak systolic velocity, cm/s	23.4 ± 1.7	24.1 ± 2.3	20.4 ± 2.0
	Pulsatility index	1.24 ± 0.11^b^	1.63 ± 0.13^a^	1.42 ± 0.13^a,b^
	Heart rate, bpm	60 ± 3	52 ± 3	56 ± 3

*The infusion treatments were reversed between the two phases in Experiment 2. Goats were fed *ad libitum* chopped orchardgrass–timothy grass hay and baseline measures for Experiment 2 were obtained during the adaptation period when the goats were fed the hay prior to initiating the infusion treatments*.

*^a,b^Measures within rows with different superscripts are different (*P* ≤ 0.05)*.

### Experiment 2

**Figure 3:**
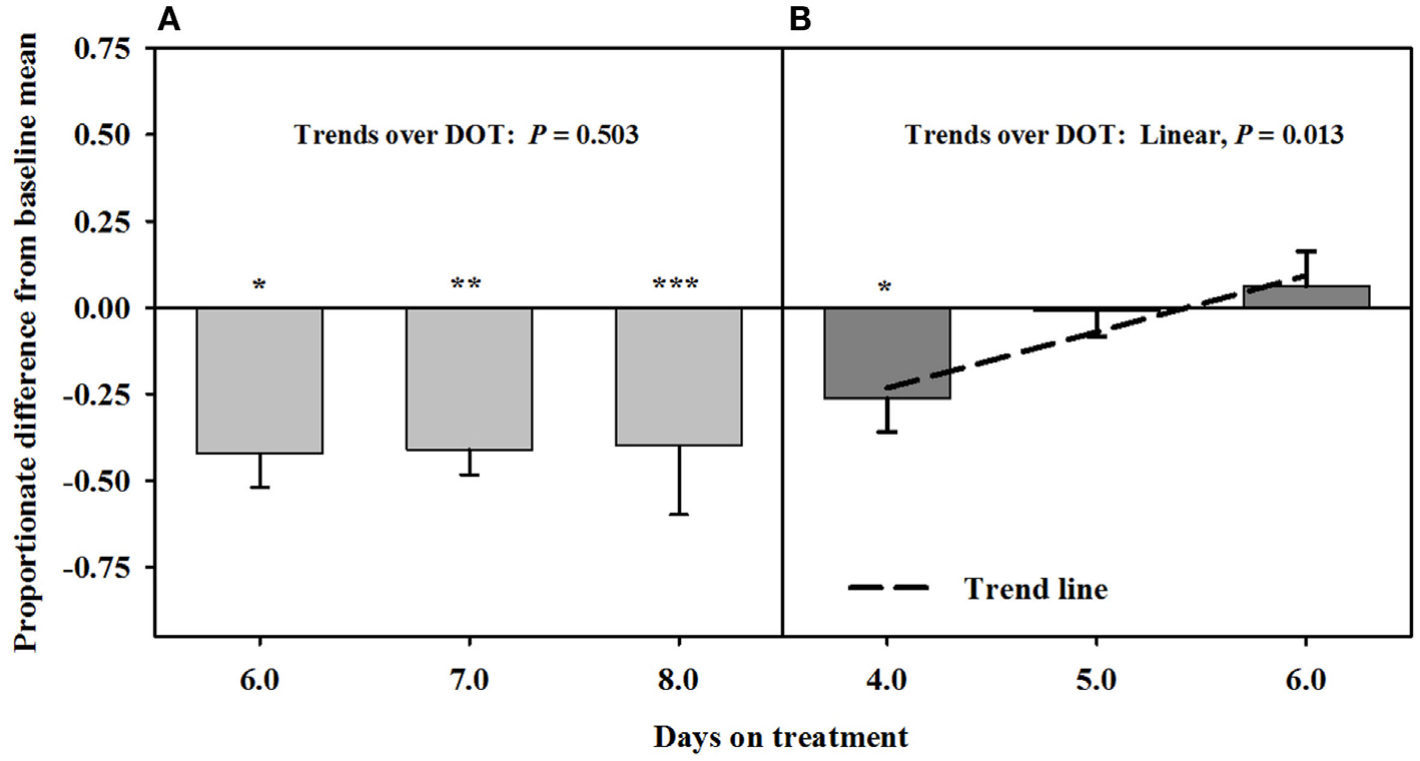
**Ultrasonic measures in Experiment 2 of proportionate differences from baseline measures for luminal area of the carotid artery in rumen-fistulated wether goats that were infused with (A) toxic seed extract only in phase A, and (B) toxic seed extract and isoflavones in phase B**. Baseline measures were taken with goats receiving *ad libitum* chopped orchardgrass (*Dactylis glomerata*)–timothy (*Phleum pratense*) hay with no rumen infusions. Images for determining baseline measures were collected when goats were being adapted to the hay diet on the last, fourth, and fifth days prior to initiating the infusion treatments. Asterisks above the bars denote significant differences between treatment and baseline measures at *P* < 0.05 (*), *P* < 0.01 (**), and *P* < 0.001 (***) levels of significance. A trend line is provided for Phase B that illustrates the linear relationship between proportionate differences from baseline and days on treatment.

After being infused with the seed extract for 6 days in Phase A, mean carotid luminal areas were proportionately less than baseline measures (14.5 ± 1.0 mm^2^) to the same extent over 6, 7, and 8 DOT (Figure 3), averaging ~41% decrease in area relative to the baseline. Carotid luminal area was still less than the baseline on day 4 after including the isoflavones with the seed extract; however, the areas increased linearly over DOT and were similar to baselines on 5 and 6 DOT.

**Figure 4:**
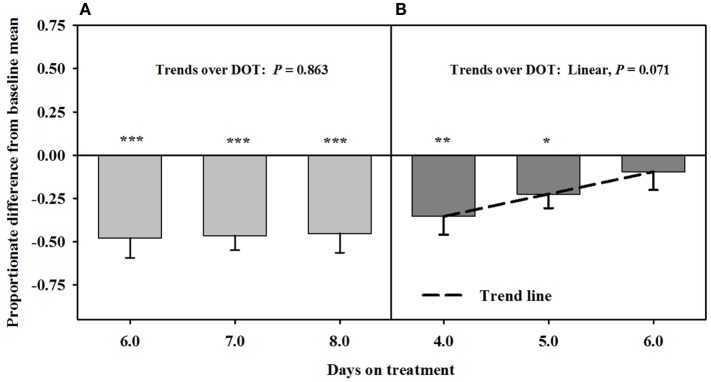
**Ultrasonic measures in Experiment 2 of proportionate differences from baseline measures for blood flow rate through the carotid artery in rumen-fistulated wether goats that were infused with (A) toxic seed extract only in phase A, and (B) toxic seed extract and isoflavones in phase B**. Baseline measures were taken with goats receiving *ad libitum* chopped orchardgrass (*Dactylis glomerata*)–timothy (*Phleum pratense*) hay with no rumen infusions. Images for determining baseline measures were collected when goats were being adapted to the hay diet on the last, fourth, and fifth days prior to initiating the infusion treatments. Asterisks above the bars denote significant differences between treatment and baseline measures at *P* < 0.05 (*), *P* < 0.01 (**), and *P* < 0.001 (***) levels of significance. A trend line is provided for Phase B that illustrates the linear relationship between proportionate differences from baseline and days on treatment.

Blood flow rate in the carotid artery responded similarly as luminal area of the artery (Figure 4). Flow rates were proportionately less than the baseline measures after fescue seed extract infusion (112 ± 10 mL/min.) over 6, 7, and 8 DOT, and the areas averaged ~47% of baseline areas. Flow rates increased linearly over 4, 5, and 6 days after isoflavones were included in the infusion treatment and were similar to baseline by 6 DOT.

**Figure 5:**
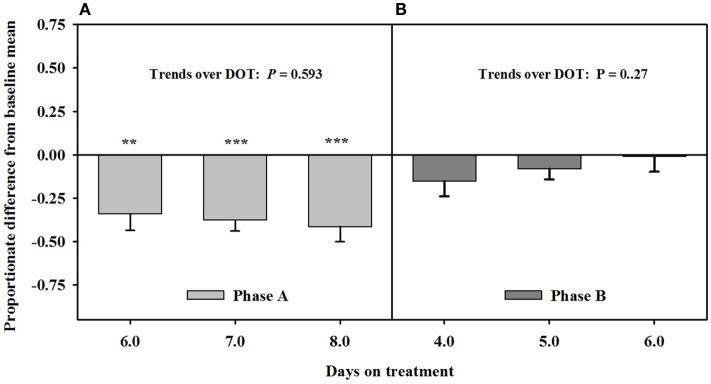
**Ultrasonic measures in Experiment 2 of proportionate differences from baseline measures for luminal area of the interosseous artery in rumen-fistulated wether goats that were infused with (A) toxic seed extract only in phase A, and (B) toxic seed extract and isoflavones in phase B**. Baseline measures were taken with goats receiving *ad libitum* chopped orchardgrass (*Dactylis glomerata*)–timothy (*Phleum pratense*) hay with no rumen infusions. Images for determining baseline measures were collected when goats were being adapted to the hay diet on the last, fourth, and fifth days prior to initiating the infusion treatments. Asterisks above the bars denote significant differences between treatment and baseline measures at *P* < 0.05 (*), *P* < 0.01 (**), and *P* < 0.001 (***) levels of significance.

Mean luminal area of the interosseous artery also was stable and less than baseline measures (0.7 ± 0.1 mm^2^) over 6, 7, and 8 DOT when only the seed extract was infused (Figure 5). Decreases in interosseous luminal areas ranged from 34.2 to 41.3% of the baseline measures. By 4 DOT after isoflavones were included with the seed extract in the infusion, the luminal areas had increased and stabilized to areas that were similar to baseline measures.

Peak systolic and mean velocities in the carotid artery did not vary (*P* = 0.889) over DOT for the infusion treatments, and were similar (*P* = 0.338) between baseline measures and both infusion treatments (Table 1). Heart rate also did not differ (*P* = 0.339) between the baseline and infusion treatments. Pulsatility index with the seed extract infusion was greater (*P* = 0.025) than the baseline measure, but decreased back to indices that were similar (*P* = 0.25) to the baseline average when isoflavones were included with seed extract in the infusion.

## Discussion

The focus of this research was to investigate if isoflavones can mitigate the adverse effects of ergot alkaloids on the hemodynamics of goats. Persistent constriction of blood flow to peripheral tissues incapacitates the animal’s ability to thermoregulate their core body temperature. This causes animals being vulnerable to severe heat stress under moderate ambient temperatures. Recent research has reported ergot alkaloid-induced vasoconstriction of the ruminal artery and vein (27), and mesenteric vasculature (28) in cattle. These results provided strong evidence that exposure to ergot alkaloids can have a negative influence on nutrient efficiency.

Low prolactin concentrations are often used as an indicator that animals are suffering from fescue toxicosis. It would have been informative to monitor serum prolactin concentrations in the present experiment with goats; however, it was critical to control goat temperament and nervousness for accurate measurement artery lumen areas and blood flow characteristics. Although body temperature measures and collection of blood for assaying prolactin concentrations would have been informative, they would likely have caused extraneous error in the ultrasound measures that were necessary in meeting experimental objectives.

The diet concentrations of isoflavones used in the experiments were based on the concentration needed to decrease ammonia production *in vitro* (24). However, it should be mentioned that selectivity of clovers relative to grasses and other forages could be such that typical diet concentrations of isoflavones are much lower or higher than what is used in the present experiment. Dose–response experiments and grazing experiments with clover–grass mixtures will be needed to determine optimum diet concentrations of isoflavones for mediation of a response.

### Experiment 1

Ergovaline is the ergopeptine that is consistently in the highest concentration in toxic endophyte-infected tall fescue (29) and also has demonstrated to be the most potent ergopeptine produced by the tall fescue endophyte in mediating a vasoconstrictive response in bovines (30, 31). Aiken et al. (32) reported a vasoconstrictive response of the caudal arteries of endophyte-naïve heifers in less than 48 h after initial consumption of diets containing ground tall seed in quantities that provided ergovaline concentrations of 0.8 μg/g DM.

Carotid and auricular arteries in goats were reported by Aiken and Flythe (11) to exhibit a vasoconstrictive response in 2 days after initial consumption of a diet containing 0.8 μg ergovaline + ergovalinine/g DM. Responses in Experiment 1 were similar between the carotid and interosseous arteries. A diet concentration similar to that used by Aiken and Flythe (11) was fed in the preset experiment, but alkaloid-induced vasoconstriction did not occur over the first 4 days when rumens were infused with both toxic seed and isoflavones. There did, however, appear to be vasodilation of the carotid artery at 3 and 4 DOT. The strong linear decline in luminal areas after isoflavones were removed from the infusion provided evidence that isoflavones mediated vaso-relaxation of the carotid artery. Similar to the findings of Aiken and Flythe (11), significant vasoconstriction occurred at 2 DOT when only the toxic seed was infused.

Higher pulsatility reflects greater resistance to blood flow; thus, greater blood flow resistance was observed on 4 DOT with infusions lacking isoflavones. Greater peak systolic velocity could also be expected with higher pulsatilities, but there can be considerable error in these measures due to between-animal and between-image variations. Aiken and Strickland (33) warned that placement of the sample gate within the vessel and animal tension could be major sources of variation between images.

### Experiment 2

Based on reduced luminal areas of both arteries at 6, 7, and 8 DOT during phase A when only the seed extract was infused, the arteries appeared to have been saturated with ergot alkaloids. This is because the luminal areas at each of these latter DOT were less than the baselines and with no declining trends. Furthermore, blood flow rates in the carotid were similar to the responses for the luminal areas to seed extract over DOT. Luminal area and blood flow rate for the carotid were less than baselines on 4 DOT, but linear increases over the latter DOT indicated that the areas and blood flows were back to baseline levels by 5 DOT with inclusion of isoflavones in the infusion. Luminal areas of the interosseous artery did not exhibit increases relative to the baseline, but were similar to baseline at each of the three DOT; therefore, vaso-relaxation had occurred prior to 4 DOT.

The responses of the carotid arteries to isoflavones appeared to be gradual during the phase; however, measures were needed at earlier DOT to verify that trend. Again, saturation of the carotid was indicated to have occurred in Phase A, which could have been a factor in early mitigation of the vasoconstriction. The mitigation of vasoconstriction of the interosseous artery might have occurred earlier than 4 DOT, which suggests that there could be a greater density of β-adrenergic receptors in the interosseous than carotid artery.

It is curious that baseline measures of mean luminal area of the interosseous were almost fourfold greater in Experiment 1 than in Experiment 2. This was likely related more to the methods of measuring luminal area and not to differences in accuracy between the two ultrasound instruments, which was checked. Aiken et al. (8) concluded that tracing connective tissue in the intima with a B-mode image could overestimate luminal area and tracing the color Doppler signal could underestimate the signal. However, it was difficult without using the color Doppler signal as a guide to take the measures at the true peak systolic phase of the cardiac cycle because of a weaker pulse of the interosseous artery, as compared to the carotid artery. Tracing the color Doppler signal offers more accuracy in taking measures at the peak velocity during a cardiac cycle, whereas relying on weaker pulses without color Doppler could cause errors in detection of peak systolic velocity that would lead to underestimation of luminal area.

Peak systolic velocity and heart rate were not affected by either infusion treatment; however, the least square mean for pulsatility index was greater for the seed extract without than with isoflavones, and the baseline measure. Similar mean pulsability indices between the baseline and the seed extract and isoflavone infusion further indicated that isoflavones mitigated resistance to blood flow mediated by the alkaloid-induced vasoconstriction.

Jia et al. (34) examined the interaction of the isoflavones, formononetin and biochanin A, and tall fescue seed extract on the vasoactivity of bovine mesenteric arteries and veins. In these experiments, the two isoflavones tested did not mitigate vasoconstriction, which superficially appears to contradict the results presented here. However, comparison of the studies is informative. The most obvious difference is that the experiment performed by Jia and co-workers was *in vitro*, on a myograph, and with different blood vessels than those monitored in the current study. It is well established that β-adrenergic receptors are differentially expressed in tissue types across species (35, 36). Thus, isoflavones could have greater impact on some regions of the vascular system than others, which is consistent with the activity of known β-adrenergic agonists, such as ractopamine (37). Second, the mesenteric arteries and veins were exposed to the isoflavones for 2 h prior to the myograph measurements, while the goats received the isoflavones in the rumen daily. Microorganisms metabolize some isoflavones in the rumen (38). For example, formononetin is converted into daidzein and then equol, which undergoes renal clearance (39, 40). The detection of equol in the urine indicates that equol, rather than formononetin, was present in the blood.

When the results of the current study and the results of Jia and co-workers are considered together several plausible hypotheses emerge, including (1) clover isoflavones mitigate vasoconstriction in the carotid and interosseous arteries, but not in the mesenteric artery and vein; (2) exposure periods longer than 2 h are required; and (3) the biologically active forms are microbial metabolites of the clover isoflavones. Differences in animal species should also be considered, but previously demonstrated activities in a variety of animals (including humans and rodents) make differences between the two ruminant species the less likely possibility (16, 19, 12). It is also possible that the amounts of isoflavones in the fed tablets were underestimated. The amounts determined in this study were about 25% of the total isoflavone content reported on the product label. The discrepancy may reflect differences in the extraction or the quantitation methods used. It may also be due to the isoflavone extraction employing 30 min of sonication at ambient temperature instead of refluxing (15 min) at 85°C, the method used by Krenn et al. (41). Sonication for 30 min in acidified methanol has thoroughly extracted phenolic compounds from fruits and vegetables in other studies (42). However, it is possible that the sonication did not completely extract isoflavones from the clover tissue. In that case, the amount of isoflavones available to the animal may have exceeded the amounts determined by the tablet analysis, although it is difficult to determine how the amounts extracted by acidified methanol would compare to the amounts extracted by rumen microorganisms in an aqueous environment.

## Conclusion

Results indicated that isoflavones in the diet of goats grazing toxic endophyte-infected tall fescue can counter and relieve ergot alkaloid-induced vasoconstriction. Although results from Experiment 2 indicated that relief with isoflavones can occur after goats are exposed to ergot alkaloids, it is uncertain if the vasculature of the goats was saturated with ergot alkaloids, which could have had a bearing on efficacy of the treatment. More experiments are needed to verify the responses and evaluate dose–responses for determining threshold concentrations of isoflavones in the diet for mediating a positive response by the vasculature. Furthermore, the rapidness of the responses to both ergot alkaloids and isoflavones were such that valid interpretations and conclusions could be made using Latin square types of experimental designs, which will be necessary if environmental controls are not possible. Nonetheless, these results provide strong evidence that isoflavones produced by legumes can ameliorate the adverse effects of fescue toxicosis.

Studies with cattle, sheep, and horses should be conducted to determine if alkaloid-induced vasoconstriction can be relaxed with exposure to isoflavones. Given that isoflavones ameliorate alkaloid-induced vasoconstriction in grazing livestock, management approaches can be developed to utilize isoflavone containing clovers and other legumes in pastures or feed additives to alleviate or mitigate severe heat stress suffered by animals grazing toxic tall fescue in moderate air temperatures. Relaxation of alkaloid-induced vasoconstriction from consumption of isoflavones could alleviate or mitigate the adverse effects of ergot alkaloids on blood circulation and the ability to thermos-regulate core body temperature. This could ultimately enhance dry matter intake and improve overall animal performance and well-being. However, findings from the present experiments should be verified with replicated grazing trials that use a higher number of animals and with longer term exposure to ergot alkaloids.

## Author Contributions

GA conceived the research, designed and planned the experiment, conducted ultrasonography measures, analyzed data, and wrote the manuscript. MF assisted in the design and planning of experiment, performed the infusion treatments, and assisted in writing the manuscript. IK conducted analyses of biochanin A and determined the diet concentrations. Also, assisted in the writing of the manuscript. HJ conducted analyses of ergovaline in seed and seed extracts. LB provided the seed extracts and assisted in writing the manuscript.

## Disclaimer

Mention of trade names or commercial products in the article is solely for the purpose of providing specific information and does not imply recommendation or endorsement by the USDA. The United States Department of Agriculture is an Equal Employment Opportunity Employer.

## Conflict of Interest Statement

The authors declare that the research was conducted in the absence of any commercial or financial relationships that could be construed as a potential conflict of interest.
